# Global trends and hotspots of gastrointestinal microbiome and toxicity based on bibliometrics

**DOI:** 10.3389/fmicb.2023.1231372

**Published:** 2023-07-31

**Authors:** Jiajia Duan, Chuanxin Liu, Xiaoyang Bai, Xiaoying Zhao, Tao Jiang

**Affiliations:** ^1^Department of Clinical Laboratory, The First Affiliated Hospital, and College of Clinical Medicine of Henan University of Science and Technology, Luoyang, China; ^2^Medical Key Laboratory of Hereditary Rare Diseases of Henan, Luoyang Sub-Center of National Clinical Research Center for Metabolic Diseases, Endocrine and Metabolic Disease Center, Department of Metabolism and Endocrinology, The First Affiliated Hospital, and College of Clinical Medicine of Henan University of Science and Technology, Luoyang, China; ^3^Department of Medical Equipment, The First Affiliated Hospital, and College of Clinical Medicine of Henan University of Science and Technology, Luoyang, China; ^4^The Second Ward of Department of Digestive Oncology, The Sixth People’s Hospital of Luoyang, Luoyang, China

**Keywords:** gastrointestinal microbiome, toxicity, bibliometrics, CiteSpace, VOSviewer

## Abstract

**Background:**

Toxicity concerns persist in the fields of public health, environmental science, and pharmacology. The intricate and vital role of the gastrointestinal microbiome in influencing toxicity and overall human health has gained increasing recognition in recent years. This study presents a comprehensive bibliometric analysis to evaluate the global scientific output, emerging trends, and research focal points in the area of gastrointestinal microbiome and toxicity.

**Methods:**

The Web of Science Core Collection database was retrieved for publications on the gastrointestinal microbiome and toxicity from 1980 to 2022. Our analysis included scholarly research papers written in English and excluded duplicate publications. We used Biblioshiny and R to summarize the count and citation metrics of included articles, and visualized research trends and keywords. CiteSpace was used to identify reference literature, keywords, and citation bursts. VOSviewer was used to visualize the network of related countries, institutions, authors, co-cited authors, and keywords.

**Results:**

A total of 2,140 articles were included, allowing us to identify significant countries, institutions, authors, and research focal points. Our results indicate a growing trend in the field, with China and the United States leading the research. The most productive journal in this area is Science of the Total Environment. Key findings revealed that research hotspots have shifted from drugs to environmental pollutants, emphasizing microplastics. Important mechanisms studied include oxidative stress, metabolism, inflammation, and apoptosis, with target organs being the gastrointestinal tract, liver, and brain. Furthermore, we highlight the rising significance of the gut-brain axis and the usage of zebrafish as a model organism.

**Conclusion:**

Despite certain limitations, such as focusing solely on English-language publications and excluding unpublished literature, our findings provide valuable insights into the current state of research on toxicity and the gastrointestinal microbiome. In the future, modifications to the gastrointestinal microbiome could offer new directions for treating and mitigating toxicity. These discoveries provide a comprehensive perspective on the broader scope of this research field.

## Introduction

Toxicity issues have long been a primary concern in public health, environmental protection, and pharmacology. Toxicity research is essential for ensuring environmental, food, drug, and occupational safety and developing and evaluating new drugs (Arome and Chinedu, 2013). With the advancement of science and technology and changing societal needs, toxicity research has shifted from descriptive to mechanistic, from single substances to composite materials, from animal experiments to alternative methods, and from empiricism to system science. The gastrointestinal microbiome refers to the complex community of microorganisms in the human gastrointestinal tract, including bacteria, fungi, viruses, and more (Lindon et al., 2018). There is an increasing consensus suggesting that the gastrointestinal microbiome plays a crucial role in maintaining human health (Marchesi et al., 2016). These microorganisms participate in the body’s metabolism and absorption of nutrients and are closely associated with various physiological functions, such as the immune and endocrine systems (Belkaid and Hand, 2014; Gomaa, 2020).

The past decade has witnessed a significant surge of interest in understanding the gastrointestinal microbiome and its crucial role in maintaining health and contributing to the development of diseases. This growing interest has prompted a rapid expansion of research efforts to explore the gastrointestinal microbiome’s influence on various aspects of human wellbeing, including its potential relationship with toxicity. Concurrently, there has been an increased incidence of gastrointestinal disorders that could be influenced by the gastrointestinal microbiome and toxicity. For instance, recent estimates suggest that over 70 million people in the United States suffer from gastrointestinal (GI) disorders (O’Neill et al., 2021). The global rise in GI disorders has been linked to factors such as dietary changes, elevated stress levels, and exposure to environmental toxins, all of which can impact the gastrointestinal microbiome (Mohr et al., 2020). Given the burgeoning interest in this field and its potential implications for public health, there is a pressing need for a comprehensive and systematic analysis of the current state of knowledge, which is the primary focus of our study.

In recent years, research has unveiled complex interactions between the gastrointestinal microbiome and toxicity, which can be synergistic or antagonistic. On the one hand, the gastrointestinal microbiome can metabolize certain exogenous or endogenous toxic substances (Jeong et al., 2013), such as drugs, environmental pollutants, and endocrine disruptors, altering their structure, bioavailability, activity, or toxicity (Wilson and Nicholson, 2017). On the other hand, the gastrointestinal microbiome can also produce toxic metabolites such as D-lactic acid, ammonia, endotoxins, or carcinogens, thereby increasing the toxicity experienced by the host (Van de Wiele et al., 2005). Furthermore, an imbalance or disruption of the gastrointestinal microbiome, such as a decrease in microbial populations, overgrowth, or displacement, can lead to immune dysfunction, intestinal barrier damage, inflammatory responses, or systemic toxicity within the host (Chi et al., 2019).

Therefore, comprehending the mechanisms underlying the interactions between the gastrointestinal microbiome and toxicity, and finding ways to modulate the gastrointestinal microbiome to prevent or treat diseases associated with toxicity, are subjects of great importance and complexity. Various strategies centered around the gastrointestinal microbiome have been proposed or developed, including probiotics, fecal transplantation, microbial metabolites, and synthetic biology. These approaches promise to enhance the effectiveness and safety of drugs, reduce the risk of exposure to environmental pollutants, and augment the immune response against cancer.

Bibliometric analysis is a quantitative research method widely used in scientific research globally (Bornmann and Mutz, 2015). It has become one of the most extensively employed methods for evaluating the credibility, quality, and impact of academic work (van Raan, 1996). Bibliometric analysis can help us understand the current state, development trends, and research hotspots in a particular field. However, no bibliometric studies have hitherto been conducted on the association between gastrointestinal microbiome and toxicity. Therefore, we conducted a bibliometric analysis to reveal the major achievements, key technologies, and future development directions in this field. We hope this work can serve as a guide to identify critical knowledge and research priorities, assist researchers in formulating research strategies, optimize resource allocation, and achieve more breakthrough results in gastrointestinal microbiome and toxicity.

## Materials and methods

### Data acquisition

Bibliometric research is a quantitative method used to analyze and evaluate scientific literature. Web of Science (WOS) is one of the most commonly used academic database sources and is considered the most comprehensive and reliable database in bibliometric analysis (Wang et al., 2015). To prevent bias caused by daily database updates, the literature relevant to this study was searched for and exported from the Web of Science Core Collection (WOSCC) as plain text files containing full records and cited references on February 20, 2023.

Our search strategy details are provided in Supplementary Table 1. For this study, the literature was selected based on the following inclusion and exclusion criteria: (i) The publication timeframe was limited to articles published until December 31, 2022; (ii) Only research articles and reviews were considered for inclusion; (iii) There were no limitations on the species or organisms studied; (iv) Publications written in English were included, while those in other languages were excluded; (v) Duplicate publications were removed from the analysis to ensure the uniqueness of the dataset. Given that this study uses publicly available secondary data and does not involve interaction with human subjects, it does not require an ethical review.

### Data analysis

Our bibliometric analysis study was conducted using multiple tools, including Biblioshiny and R-version 4.2.2, VOSviewer (1.6.19), CiteSpace 6.2.R1 (64-bit), Tableau 2022.4, and Microsoft Excel 2021.

The Impact Factor (IF) is a widely used metric for assessing the significance and influence of scientific journals. It is calculated by dividing the total number of citations received by papers published in the journal during the previous 2 years by the total number of papers published in that journal during the same period (Garfield, 2006).

CiteSpace is a software tool developed by Professor Chaomei Chen of Drexel University to analyze and visualize scientific literature, particularly in the field of bibliometrics (Chen, 2017). It is widely used by researchers, librarians, and information professionals to gain valuable insights into the structure and evolution of scientific fields. In this study, we utilized CiteSpace to conduct visual analyses encompassing various aspects such as country distribution, institutional distribution, reference analysis, and keyword and citation bursts. The specific parameters used in CiteSpace were set as follows: time slicing (from 1980 to 2022, years per slice = 1), term source (title, abstract, author, keyword, and keywords plus), node type (one option chosen at a time from author, institution, country, keyword, cited reference, cited author, and cited journal), link strength (Cosine), link scope (Within Slices), selection criteria (g-index *K* = 25), and pruning (none).

VOSviewer is a powerful tool for visualizing and analyzing bibliometric networks based on distance-based bibliometric tools. It was developed by Nees Jan van Eck and Ludo Waltman of Leiden University, and can categorize related items into clusters of various colors, with the same color indicating a higher degree of association among these items (Van Eck and Waltman, 2011). This study used VOSviewer to examine and visualize the distribution of countries, institutions, authors, and co-cited authors and explore keyword co-occurrence and overlay networks.

Biblioshiny is a web-based software tool built using R programming language, designed to facilitate bibliometric analyses and generate interactive visualizations of the obtained results. It was jointly developed by Aria and Cuccurullo (2017) from the University of Naples and the University of Campania in Italy. In our study, we primarily utilized Biblioshiny to summarize the volume and citation counts of the bibliometric analyses, assess the performance of articles and journals, identify the occurrence of annual cumulative keywords/terms, calculate national influence and collaboration frequency, and visualize research trends and keyword timelines. The specific parameter information used in Biblioshiny is detailed in Supplementary Table 2. We also used generalized additive models using the MGCV package in R to estimate the trend and quantity of related literature publications (Wood, 2018). The source code file is provided in Supplementary Table 3.

Tableau is a widely used visualization tool for exploring and analyzing data. In this study, it was employed to analyze and visualize the distribution of publications over time and the number of publications by country.

## Results

### Publication output and trends

**FIGURE 1:**
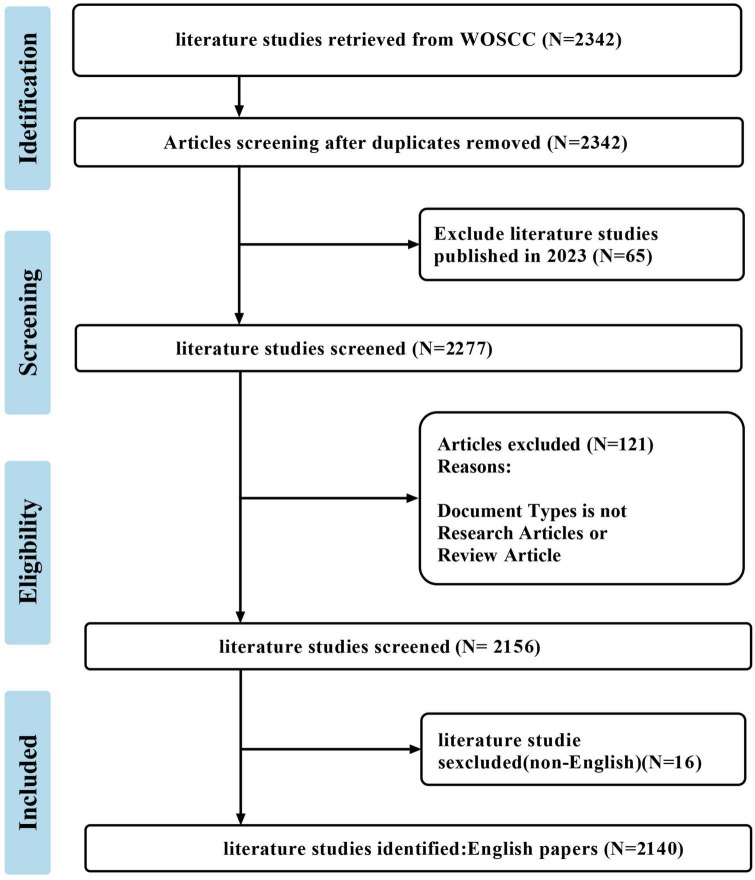
Flow diagram of the selection process.

**FIGURE 2:**
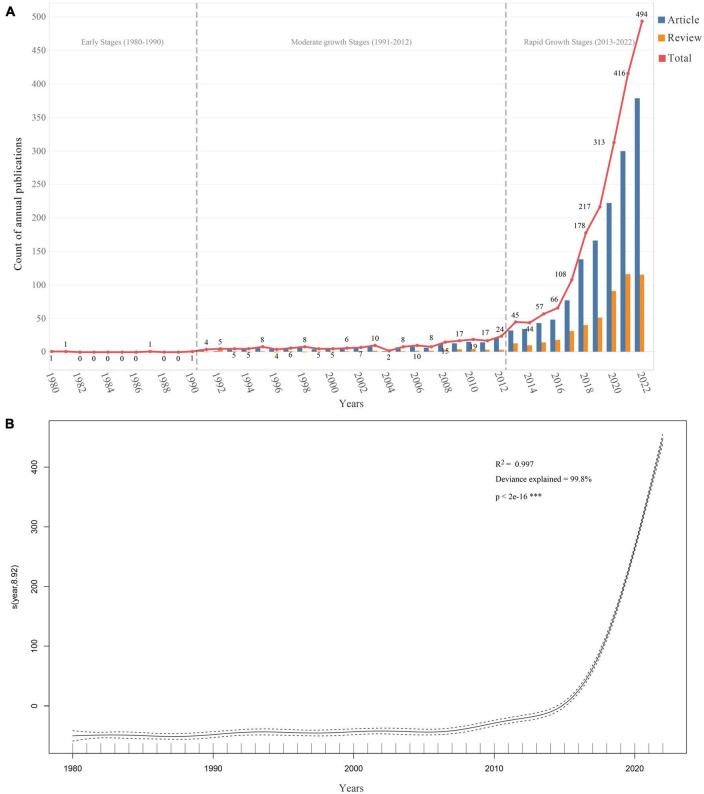
Trends in the number of publications on gastrointestinal microorganisms studied in the field of toxicity from 1980 to 2022. **(A)** The annual number of publications. **(B)** Generalized additive model fit curve plot.

After the literature retrieval and screening process, 2,140 publications met the inclusion and exclusion criteria (Figure 1), including 1,606 research articles and 534 review articles. As shown in Figure 2A, the first article was published in 1980 (Carter et al., 1980). From 1980 to 2022, the number of publications increased from 1 in 1980 to 494 in 2022, with an annual compound growth rate of 15.52%. Although the number of annual publications fluctuated slightly, an overall increasing trend was observed. To facilitate a comprehensive understanding of the development of publications in this field, the timeline was divided into three distinct stages: (i) Early stage (1980–1990): during this stage, the number of publications was extremely low, with a maximum of only 1 record per year.; (ii) Moderate growth stage (1991–2012): during this stage, the number of publications began to increase with 4–24 publications/per year; (iii) Rapid growth stage (2013–2022): during this stage, the number of publications escalated, with the annual number of articles increasing from 45 to 494 in 2022.

A generalized additive model was also used to analyze the relationship between the number of publications and the corresponding year (Figure 2B). The formula for the model is “count ∼ s(year),” where “year” is the predictor variable, and the smooth term “s(year)” indicates that the relationship between “year” and “count” is not strictly linear, and is modeled using a smoothing function. The effective degrees of freedom (edf) for the smoothing term were 8.918, and the corresponding F-statistic and *p*-value indicated that the smoothing term was highly significant (*p*-value < 2e-16). The model demonstrated a strong fit to the annual trend of publications (*R*^2^ = 0.997, Deviance explained = 99.8%). Based on the model’s prediction, the total number of publications related to the toxicity of gastrointestinal microbiome is projected to exceed 592 in 2023 and will continue to increase in the next 10 years to 2032, with an estimated 1,445 publications in 2032.

### Most prolific countries/regions analysis

**FIGURE 3:**
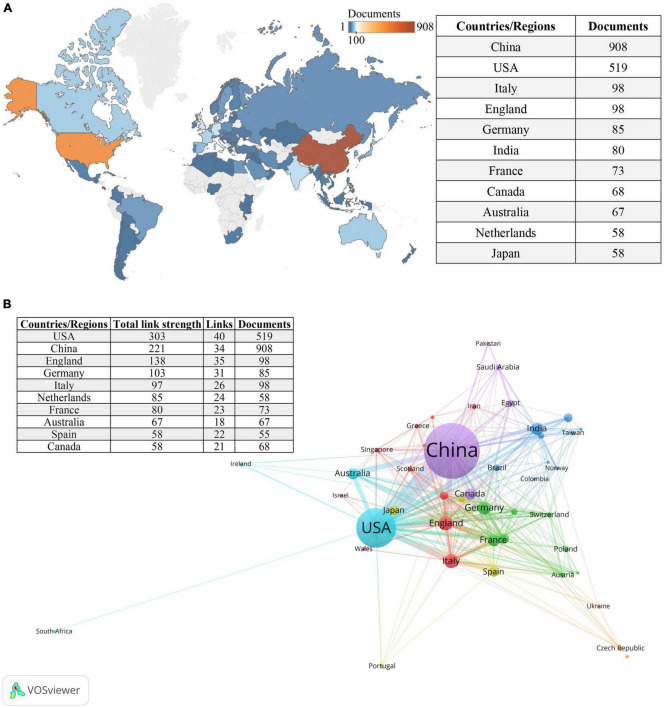
Countries/regions involved in the field of research. **(A)** World map of countries/regions based on the number of related publications. **(B)** The countries/regions collaboration network in this field. Circles represent countries, with the size of the circle indicating the number of publications. Different colors represent distinct clusters, while the lines connecting items represent international collaborations between countries. The thickness of the connecting lines indicates the strength of the collaboration.

Figure 3A provides an overview of the distribution of the 2,140 included articles among 84 countries/regions. The top 10 countries/regions, ranked by the number of publications, include a diverse representation from different parts of the world. Among these top contributors, there were five European countries, three Asian countries, two North American countries, and Australia. China emerged as the country with the highest number of publications (*n* = 908), followed by the United States (*n* = 519), while the remaining countries/regions published fewer than 100 articles each. Figure 3B illustrates the international collaborations among 47 countries/regions that have collaborated on at least five publications.

### Most prolific institutions

**FIGURE 4:**
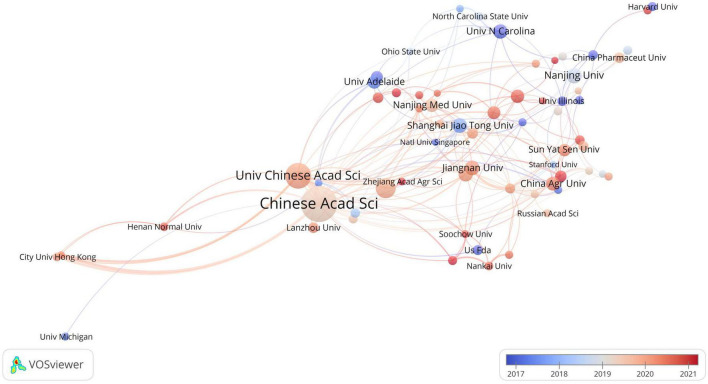
The overlay visualization of institutional collaboration in this field. Circles represent institutions, with the size of the circle indicating the number of publications. The gradient of the color denotes the average starting year of publications in a specific research area for each institution. Lines connecting the items represent collaborations between institutions, while the thickness of these lines signifies the strength of the collaboration.

The analysis revealed that a total of 2,585 institutions made contributions to research in the field of toxicology and gastrointestinal microbiome. The top 10 most productive institutions are presented in Table 1. Within the overlapping network of institutional collaboration analysis, 66 institutions published more than 10 related articles (Figure 4). Researchers affiliated with the University of London’s Imperial College of Science, Technology and Medicine, and Harvard University, have been actively engaged in this field since its early stages. On the other hand, researchers from the Chinese Academy of Fishery Sciences and Chengdu University of Traditional Chinese Medicine have more recently begun exploring this study area.

**TABLE 1:** Top 10 institutions in terms of number of articles issued.

Rank	Institution	Documents	Original country
1	Chinese Academy of Sciences	111	China
2	University of Chinese Academy of Sciences	67	China
3	Zhejiang University of Technology	46	China
4	Nanjing University	30	China
5	Jiangnan University	29	China
6	Shanghai Jiao Tong University	29	China
7	Univ N Carolina	27	USA
8	Zhejiang University	27	China
9	China Agricultural University	26	China
10	University of Adelaide	25	Australia

### Journal analysis

The 2,140 selected publications were published across 745 journals. Among them, 415 articles were published in the top 10 journals, accounting for 19.4% (415/2140) of the total articles. Table 2 presents the top 10 journals and their respective IF for 2022. Among these journals, 90% (9/10) were classified as Q1 in the Journal Citation Reports (JCR), with five journals categorized as environmental sciences and nine with an IF greater than 5. In terms of publisher location, four of the top 10 journals were based in the United Kingdom, two in the United States, two in the Netherlands, and two in Switzerland. Furthermore, we employed Bradford’s Law to identify the core journals in gastrointestinal microbiota and toxicology research (Bradford, 1934). As illustrated in Supplementary Figure 1, the application of Bradford’s Law identified 28 core journals, with one-third of the articles published in core journals.

**TABLE 2:** The top 10 most published journals in the field of gastrointestinal microbiology and toxicity research.

Ranking	Sources	Articles	Country	IF	JCR-c
1	Science Of The Total Environment	76	Netherlands	10.754	ENVIRONMENTAL SCIENCES–SCIE (Q1)
2	Environmental Pollution	58	England	9.988	ENVIRONMENTAL SCIENCES–SCIE (Q1)
3	Ecotoxicology And Environmental Safety	56	England	7.129	ENVIRONMENTAL SCIENCES–SCIE (Q1); TOXICOLOGY–SCIE (Q1)
4	Chemosphere	47	England	8.943	ENVIRONMENTAL SCIENCES–SCIE (Q1)
5	Frontiers In Microbiology	39	Switzerland	6.064	MICROBIOLOGY–SCIE (Q1)
6	Journal Of Hazardous Materials	37	Netherlands	14.224	ENGINEERING, ENVIRONMENTAL–SCIE (Q1); ENVIRONMENTAL SCIENCES–SCIE (Q1)
7	Plos One	28	USA	3.752	MULTIDISCIPLINARY SCIENCES–SCIE (Q2)
8	Frontiers In Pharmacology	27	Switzerland	5.988	PHARMACOLOGY AND PHARMACY–SCIE (Q1)
9	Food And Chemical Toxicology	24	England	5.572	TOXICOLOGY–SCIE (Q1)
10	International Journal Of Molecular Sciences	23	USA	6.208	BIOCHEMISTRY AND MOLECULAR BIOLOGY–SCIE (Q1)

IF, impact factor (2021); JCR-c, Journal Citation Reports category (2021).

### Most influential author analysis

Analysis of the authors in the included literature revealed that 11,894 authors contributed to publications related to gut microbiota and toxicity research. Table 3 shows the top 10 authors in terms of the number of published articles and co-citation frequency. Regarding the publication count, except for Kun Lu from the United States, the remaining nine authors were all from China. Yuanxiang Jin from Zhejiang University of Technology was the most prolific author (*n* = 28 articles), followed by Wei Chen and Hao Zhang from Jiangnan University (*n* = 25 articles), and Zhengwei Fu from Zhejiang University of Technology (*n* = 21 articles). Among the top 10 co-cited authors, four were from the United States, and two were from China. Notably, Yuanxiang Jin emerged as the most prolific author, having the highest count of publications and citation frequency, suggesting that he has been actively engaged in research and has achieved considerable recognition and influence within the gut microbiota and toxicity research field. Jeremy K. Nicholson from the Imperial College of Science, Technology and Medicine and Peter J. Turnbaugh from the University of California San Francisco ranked second and third regarding co-citation frequency.

**TABLE 3:** Top 10 authors in terms of number of publications and frequency of co-citations.

Rank	Author	Publications	Countries/ regions	Institutions	Author	Co-citations	Countries/ regions	Institutions
1	Jin, Yuanxiang	28	China	Zhejiang University of Technology	Jin, Yuanxiang	299	China	Zhejiang University of Technology
2	Chen, Wei	25	China	Jiangnan University	Nicholson, Jeremy K	241	England	Univ London Imperial Coll Sci
3	Zhang, Hao	25	China	Jiangnan University	Turnbaugh, Peter J	218	USA	University of California San Francisco
4	Fu, Zhengwei	21	China	Zhejiang University of Technology	Zhai, Qixiao	214	China	Jiangnan University
5	Zhao, Jianxin	20	China	Jiangnan University	Caporaso, J. Gregory	212	USA	Univ Colorado
6	Zhai, Qixiao	16	China	Jiangnan University	Ley, Ruth E	203	Germany	Max Planck Inst Dev Biol
7	Lu, Kun	16	USA	University of Georgia	Cani, Patrice D	174	Belgium	Catholic Univ Louvain
8	Liu, Yang	16	China	Henan Normal University	Robert C Edgar	166	USA	University of California, Berkeley
9	Tian, Fengwei	14	China	Jiangnan University	Stringer, Andrea M	153	Australia	University of South Australia
10	Li, Yuan	14	China	Chinese Academy of Medical	Wallace, Bret D	152	USA	University of North Carolina

**FIGURE 5:**
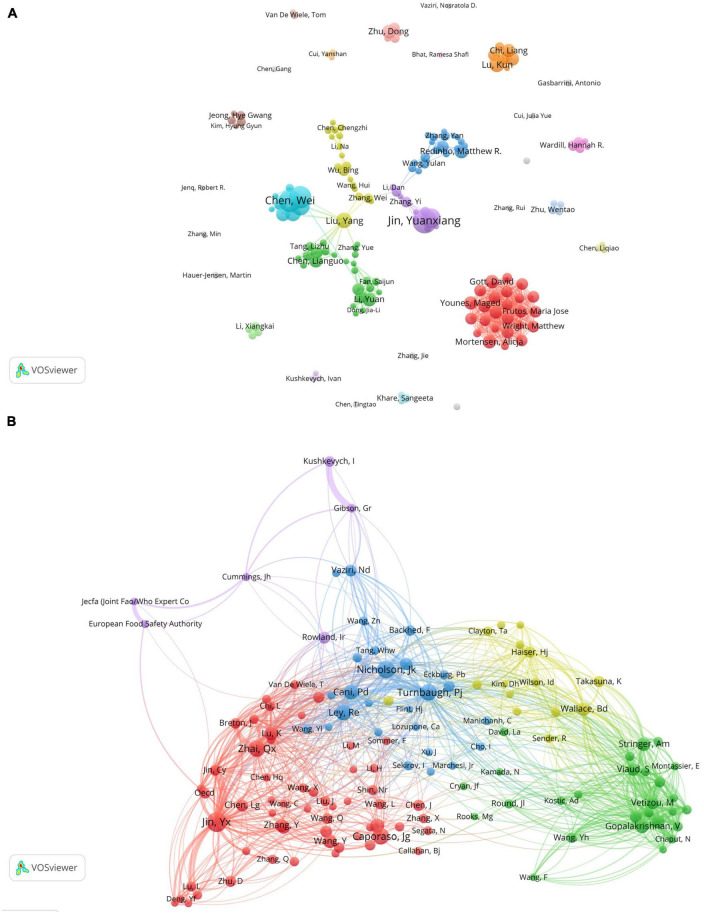
Visualization of co-authorship and co-citation relationships among authors in gastrointestinal microbiota and toxicology research. **(A)** Network visualization of collaborations between authors. The size of the items represents the number of publications by the author, and different colors mark different clusters. The thickness of the connections between items indicates the strength of the relationships. **(B)** Co-citation author network. The size of the items represents the frequency of co-citations for each author.

The author collaboration analysis was performed using VOSviewer (Figure 5A), where authors with a publication count of 5 or more were included. The 164 included authors were classified into 30 clusters based on their collaborations, and the connections between different clusters were relatively scarce, indicating limited collaboration among research teams/laboratories involved in gut microbiota and toxicity studies. Figure 5B shows the co-cited author relationship network, which included 131 authors with a citation frequency of 50 or more. The network is visualized with colored sections representing the similarities in research interests among the co-cited authors. The analysis reveals a high level of homogeneity among the focus areas of the relevant researchers, as they can be divided into 5 distinct clusters. However, it is worth noting that the purple group appears to have fewer connections with other clusters, indicating a relatively sparse relationship with the research interests of other groups.

### Analysis of research hotspots

#### Most cited local literature

The impact of an article in a particular research field can be gauged by the number of citations it receives (Sun et al., 2022). Table 4 lists the top 10 most globally cited documents. Half of these 10 articles have been cited more than 500 times, including 3 research articles and 7 review articles. Specifically, the most cited article in this field, “Plastic and Human Health: A Micro Issue?,” published in 2017, has been cited 1,042 times (Wright and Kelly, 2017). This influential review utilized interdisciplinary literature to discuss and evaluate the potential impact of microplastics on human health, highlighting that microplastics can damage human health by triggering immune responses through gastrointestinal microbiome and inflammatory reactions. The second and third most cited articles were “Symbiotic gut microbes modulate human metabolic phenotypes” (Li et al., 2008) and “Gut microorganisms, mammalian metabolism, and personalized health care” (Nicholson et al., 2005), with 809 and 674 citations, respectively.

**TABLE 4:** Top 10 most global cited documents.

Rank	Title	Author	Year	Cited	Article type	Journal	IF
1	Plastic and Human Health: A Micro Issue?	Wright, Stephanie L	2017	1,042	Review	Environmental Science and Technology	11.357
2	Symbiotic gut microbes modulate human metabolic phenotypes	Li, Min	2008	809	Article	Proceedings of The National Academy of Sciences of The United States of America	12.779
3	Gut microorganisms, mammalian metabolism and personalized health care	Nicholson, J K	2005	674	Review	Nature Reviews Microbiology	78.297
4	Baseline gut microbiota predicts clinical response and colitis in metastatic melanoma patients treated with ipilimumab	Chaput, N	2017	604	Article	Annals of Oncology	51.769
5	Environmental exposure to microplastics: An overview on possible human health effects	Prata, Joana Correia	2020	524	Review	Science of The Total Environment	10.753
6	Intestinal alkaline phosphatase detoxifies lipopolysaccharide and prevents inflammation in zebrafish in response to the gut microbiota	Bates, Jennifer M	2007	499	Article	Cell Host and Microbe	31.316
7	Postinfectious Irritable Bowel Syndrome	Spiller, Robin	2009	485	Review	Gastroenterology	33.883
8	Microbiota: a key orchestrator of cancer therapy	Roy, Soumen	2017	478	Review	Nature Reviews Cancer	69.8
9	Commensal Clostridia: leading players in the maintenance of gut homeostasis	Lopetuso, Loris R	2013	453	Review	Gut Pathogens	5.324
10	The microbiome, cancer, and cancer therapy	Helmink, Beth A	2019	448	Review	Nature Medicine	87.241

IF, impact factor (2021).

#### Cluster analysis of co-cited references

Co-citation is a research method to measure the degree of relationship between cited references, which requires two different articles to be cited by the same document, forming a co-citation relationship (Small, 1973). Table 5 shows the top 10 highly cited references in the field of gastrointestinal microbiome and toxicity research. These articles are often regarded as fundamental to the development of this research area. Among the top four ranked articles, three were published in *Science* and focused on the relationship between the gastrointestinal microbiome and anti-PD-1 immunotherapy.

**TABLE 5:** Top 10 highly cited references.

Rank	Title	Author	Year	Cited	Journal	IF
1	QIIME allows analysis of high-throughput community sequencing data	J Gregory Caporaso	2010	144	Nature Methods	47.990
2	Gut microbiome influences efficacy of PD-1-based immunotherapy against epithelial tumors	Bertrand Routy	2018	118	Science	63.714
3	Gut microbiome modulates response to anti-PD-1 immunotherapy in melanoma patients	V Gopalakrishnan	2018	117	Science	63.714
4	Commensal Bifidobacterium promotes antitumor immunity and facilitates anti-PD-L1 efficacy	Ayelet Sivan	2015	115	Science	63.714
5	Anticancer immunotherapy by CTLA-4 blockade relies on the gut microbiota	Marie Vétizou	2015	111	Science	63.714
6	The intestinal microbiota modulates the anticancer immune effects of cyclophosphamide	Sophie Viaud	2013	107	Science	63.714
7	Alleviating cancer drug toxicity by inhibiting a bacterial enzyme	Bret D Wallace	2010	107	Science	63.714
8	An obesity-associated gut microbiome with increased capacity for energy harvest	Peter J Turnbaugh	2006	102	Nature	69.504
9	Commensal bacteria control cancer response to therapy by modulating the tumor microenvironment	Noriho Iida	2013	92	Science	63.714
10	Effects of environmental pollutants on gut microbiota	Yuanxiang Jin	2017	91	Environmental Pollution	9.988

IF, impact factor (2021).

**FIGURE 6:**
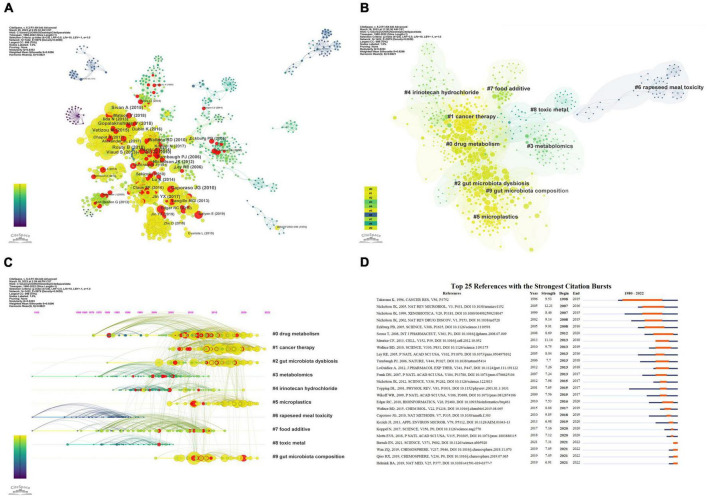
Co-citation analysis and citation burst ranking of references. **(A)** Co-citation network of references. Nodes with different colors represent different years; node size indicates citation frequency, and red nodes denote documents with citation bursts. **(B)** Top 10 clusters of co-cited references in the network. **(C)** Timeline view of co-cited references. Clusters are placed vertically in descending order of size. The position of nodes on the horizontal axis indicates the time when they were first cited, and connecting lines represent co-citation relationships. The number of citations determines the size of the nodes. **(D)** Top 25 references with the highest citation burst strength. Blue lines represent the timeline, while the orange segments denote the citation burst periods, displaying the starting year, ending year, and duration of the burst.

Furthermore, we analyzed the relationships between co-cited documents using CiteSpace 6.2.R1, and the co-citation network of cited references consisted of 1,422 nodes and 5,979 links (Figure 6A). Cluster analysis generated 192 co-citation clusters, with the top ten clusters shown in Figure 6B (Chen, 2020). The largest cluster was #0, characterized by the keyword “drug metabolism,” with the most representative article being “Effects of single and combined toxic exposures on the gut microbiome: current knowledge and future directions” by Tsiaoussis et al. (2019). Figure 6C presents a timeline view of the co-citation clusters of cited references, reflecting the temporal characteristics of research hotspots in this field.

#### Citation bursts analysis of references

Citation bursts refer to sudden increases in the number of times an article is cited, indicating heightened attention to the topic by scholars and reflecting new trends and hotspots in the field. Figure 6D presents the top 25 cited references with the strongest citation bursts. The shortest burst duration was 1 year, while the longest lasted 18 years. Among these articles, the one with the strongest citation burst was “Gut microorganisms, mammalian metabolism and personalized health care,” published in *Nature Reviews Microbiology*, with a burst spanning from 2007 to 2016 (Nicholson et al., 2005). In addition, several articles have experienced citation bursts in the past 5 years and are still ongoing, such as “The microbiome, cancer, and cancer therapy” (Helmink et al., 2019) and “Accumulation of different shapes of microplastics initiates intestinal injury and gut microbiota dysbiosis in the gut of zebrafish” (Qiao et al., 2019). These results suggest that these research directions have gained considerable popularity in recent years and are expected to continue to be a prominent area of study in the coming years.

### Keyword analysis

**FIGURE 7:**
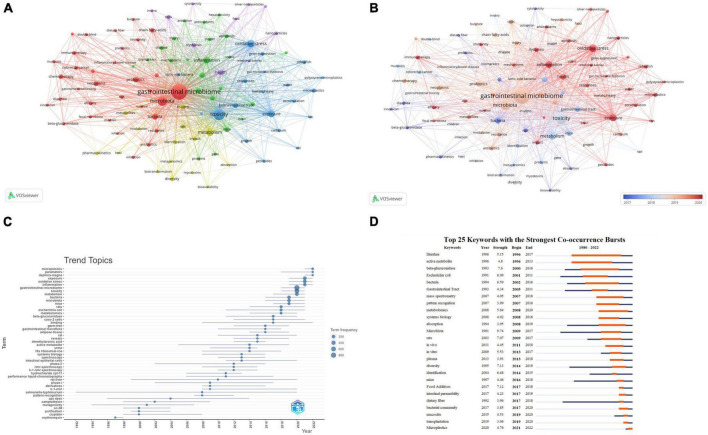
Keyword analysis. **(A)** Keyword co-occurrence network relationship diagram. Items represent keywords, and the size of items represents the frequency of keyword co-occurrence, clusters are marked using different colors, and links represent co-occurrence between keywords. **(B)** Overlay visualization of Keyword co-occurrence network. Items represent keywords; the size of items indicates the frequency of keyword co-occurrence. Early keywords are displayed in blue, while orange indicates recent keywords. **(C)** Trends in Keywords Plus development over the years. The blue line indicates the timeline of keywords, and the bubble size indicates the frequency of keywords. **(D)** Top 26 keywords with the strongest Co-occurrence frequency burst. The blue bars represent the time period when the keywords appear; the orange bars represent the time interval when the keywords are found to erupt, indicating the beginning year, end year, and duration of the outbreak.

A total of 9,628 keywords were initially considered during the analysis of keywords. To ensure the inclusion of relevant keywords while avoiding overly general or infrequent ones, a minimum occurrence threshold of 30 was applied. This resulted in 101 keywords meeting the criteria and being included in the analysis. The chosen threshold has been validated in previous bibliometric studies and has proven effective (Sweileh, 2019; Sun et al., 2022). The National Library of Medicine’s Medical Subject Headings (MeSH) vocabulary was used to consolidate similar keywords. This vocabulary is widely recognized and comprehensive in biomedical research (Supplementary Table 4). Table 6 lists the top 10 most frequently occurring keywords. Excluding those related to microbiology, the highest-frequency keywords were “toxicity,” “oxidative stress,” “metabolism,” “exposure,” “inflammation,” and “health.” Figure 7A displays the network visualization of these keywords. We observed that in the largest red cluster, the keywords were related to microbiology and cancer, such as “gastrointestinal microbiome,” “microbiota,” “neoplasms,” and “chemotherapy.” The second green cluster was associated with inflammation and metabolomics, featuring keywords like “inflammation,” “expression” and “metabolomics.” The third blue cluster was related to environmental toxicity, with primary keywords including “toxicity,” “exposure,” “microplastics,” “pesticides” and “metals, heavy.” Additionally, Figure 7B displays the overlay visualization of the keywords. The results revealed that “pharmacokinetics,” “diarrhea,” “Escherichia coli,” “cytotoxicity” and “inflammatory bowel disease” were the major themes in the early stages. In contrast, recent years have seen research hotspots focusing on keywords such as “oxidative stress,” “exposure,” “immunotherapy,” “dysbiosis,” and “microplastics.” Figure 7C illustrates the development trends of keywords over the years. As shown in Figures 7B, C we observed a gradual transition in the research focus of the gastrointestinal microbiome and toxicity fields, shifting from a focus on drugs to environmental pollutants.

**TABLE 6:** Top 10 keywords in terms of frequency of occurrence.

Rank	Keyword	Occurrences	Total link strength
1	Gastrointestinal microbiome	1,319	4,891
2	Toxicity	663	2,723
3	Microbiota	412	1,780
4	Oxidative stress	267	1,187
5	Metabolism	263	1,103
6	Exposure	234	1,099
7	Inflammation	200	938
8	Bacteria	170	757
9	Health	157	725
10	Probiotics	143	669

Moreover, we conducted a burst analysis of keywords using the CiteSpace software. Figure 7D displays the top 26 keywords with the highest burst strength. Among them, the keywords “Diarrhea” (1996–2017), “active metabolite” (1996–2013), “beta-glucuronidase” (2000–2016), and “bacteria” (2002–2016) attracted attention for an extended duration. The keyword “Microplastics” (2021–2022) was most recently used, suggesting that this keyword has recently garnered significant attention and may become a future research hotspot.

## Discussion

This bibliometric analysis investigated research development in the gastrointestinal microbiome and toxicity from 1980 to 2022. The advancement of scientific systems is closely intertwined with the progress of human civilization, and the field of toxicology is no exception. The late 20th century witnessed a golden era of development, attributable to factors such as rapid global economic growth, ecological degradation, increased exposure to human health issues, and accelerated advancements in computer science and medicine (Watson and Wexler, 2009). During this period, molecular research techniques played a crucial role in the study of gastrointestinal microbiome, resulting in significant breakthroughs in the analysis of gastrointestinal microbiome polymorphism and conducting qualitative and quantitative studies. On the other hand, the development of model organisms and genetically engineered animals has been paralleled by increased research on the physiological functions of gastrointestinal microbiome based on animal models (Stappenbeck et al., 2002; Bäckhed et al., 2004; Rakoff-Nahoum et al., 2004). In the late 20th and early 21st centuries, researchers increasingly focused on understanding the contribution of the gastrointestinal microbiome to overall health and disease states (Cho and Blaser, 2012). These early studies provided the groundwork for more complex explorations into the relationships between the gastrointestinal microbiome, disease progression, and responses to toxins.

With the official launch of the Human Microbiome Project in 2007 [ Integrative HMP (iHMP) Research Network Consortium, 2019] and the thriving development of high-throughput sequencing technologies (Caporaso et al., 2010), the scientific community’s enthusiasm for gastrointestinal microbiome research reached unprecedented heights. A review by Sekirov et al. (2010) examined the gastrointestinal microbiome’s influence on host physiology and its role in health and disease. They explored how changes in the microbiome can lead to disorders like inflammatory bowel disease and obesity, setting a foundation for further research into the microbiome’s role in disease development (Sekirov et al., 2010). In the first half of 2012, *Science* published a special issue on “The Gut Microbiota” and *Nature* followed suit in the second half of the same year with a special issue on “Gut microbes and health” (Lupp et al., 2012; Mueller et al., 2012). Since 2010, there has been a growing focus on the gastrointestinal microbiome’s role in influencing the efficacy of various treatments, including cancer therapies (Moayyedi et al., 2010; Simrén et al., 2013; Perez-Chanona and Jobin, 2014; Rajagopala et al., 2017). The results of these studies have expanded our understanding of how the gastrointestinal microbiome can modulate treatment responses, including potential toxicities. Since 2013, research on the relationship between toxicology and the gastrointestinal microbiome has gained momentum, driven by contemporary demands. Amidst this research boom, the field has amassed considerable findings and data. A comprehensive summary and analysis of its developmental trends, disciplinary frontiers, and research hotspots are important for facilitating further in-depth investigations.

China and the United States emerged as the leading countries in terms of the number of publications in the field of gastrointestinal microbiome and toxicity research, along with the highest frequency of collaboration. The substantial volume of articles from China can be attributed to increased research investment, particularly in the biomedical field, by the Chinese government in recent years (Xie and Freeman, 2019). China’s rapid economic development has also led to a heightened focus on ecological and public health issues. In terms of total link strength, the United States ranked first, primarily due to its high reputation in scientific research and innovation and numerous world-renowned research institutions and universities that attract researchers and scholars from around the world (Adams, 2012). This contributes to collaboration and development in this field (Ioannidis et al., 2018). As barriers to international exchange are eliminated, it is highly conceivable that influential countries and regions will experience complementary advantages, which will significantly impact the long-term development of this field.

Although China, India, and Japan are major countries in the gastrointestinal microbiome and toxicity research, the publishers of the ten most active journals in this field are all from Western Europe and the United States, without representation from Asia. This observation highlights the importance of developing internationally influential journals in Asia. Furthermore, we found that the majority of the top 10 journals in terms of article quantity are environmental science journals, including *Science of the Total Environment, Environmental Pollution*, *Ecotoxicology and Environmental Safety*, and others. This finding indicates that gastrointestinal microbiome and toxicity research has become a significant topic in environmental science.

In bibliometric analysis, hotspots typically refer to the most active and widely followed research directions or topics within a specific field and the characteristics and changes of hotspots can be reflected through citation analysis, keywords, and other indicators (Wu et al., 2021). Citation analysis can demonstrate the academic influence of research (Breugelmans et al., 2015). Interestingly, among the top 10 most globally cited documents, “microplastics” and “cancer therapy” were recurring topics. The top-ranked article, a review published in *Environmental Science and Technology* in 2017, was cited 1,042 times (Wright and Kelly, 2017). This article suggests that exposure to microplastics may affect the intestinal microbiome by promoting the growth of certain types of bacteria and altering the balance of microbial communities, potentially leading to adverse health outcomes such as inflammation and weakened immunity. In addition, three of the top 10 explored the role of the microbiome, particularly the gastrointestinal microbiome, in cancer and cancer therapy (Chaput et al., 2017; Roy and Trinchieri, 2017; Helmink et al., 2019). The gastrointestinal microbiome can modulate the response to various forms of cancer treatment, such as chemotherapy and radiotherapy, by influencing drug metabolism, pharmacokinetics, anticancer activity, and toxicity.

Interestingly, among the top 10 most co-cited articles, the majority (70%) focused on “cancer therapy” and were published in *Science* from 2010 to 2018. Three of these articles, cited over 110 times, were all dedicated to studying how the gastrointestinal microbiome influences the efficacy of PD-1-based immunotherapy (Sivan et al., 2015; Gopalakrishnan et al., 2018b; Routy et al., 2018). Anti-PD-1 immunotherapy is a type of cancer treatment that blocks the interaction between programmed cell death protein 1 (PD-1) and its ligands (PD-L1 and PD-L2), thereby enhancing T-cell anti-tumor activity. However, this therapy can also cause various adverse events, such as immune-related colitis. The severity and frequency of these toxicities depend on the type and combination of PD-1 or PD-L1 inhibitors used (Naidoo et al., 2015). The gastrointestinal microbiome has been shown to modulate the efficacy of anti-PD-1 immunotherapy, with higher diversity and abundance of certain bacterial taxa (such as *Bifidobacterium* and *Faecalibacterium*) being associated with better prognosis and lower incidence of immune-related adverse events (Sivan et al., 2015). Possible mechanisms by which the gastrointestinal microbiome regulates anti-PD-1 immunotherapy include affecting the composition and function of immune cells, altering the expression of PD-L1 on tumor cells, and producing metabolites that regulate inflammation and immunity (Gopalakrishnan et al., 2018a; Miller and Carson, 2020). These findings demonstrate the significant role of cancer therapy in the field of gastrointestinal microbiome and toxicity research. Therefore, interventions targeting the gastrointestinal microbiome, such as probiotics, prebiotics, antibiotics, or fecal microbiota transplantation, represent a promising strategy to improve cancer treatment outcomes and reduce toxicity.

In addition, the analysis of citation bursts, keyword clustering, and keyword bursts provides valuable insights into the key focus areas in the gastrointestinal microbiome and toxicity research. Several notable characteristics that attract significant attention in this field were identified: (1) toxic substances: drugs, nanoparticles, microplastics, heavy metals, pesticides, and biotoxins; (2) target organs: gastrointestinal tract, liver, and brain; (3) mechanisms: oxidative stress, metabolism, inflammation, and cell apoptosis. It is essential to interpret the identified burst keywords with caution. While they can be instrumental in signaling new and fast-emerging research areas, they do not always correlate with the quality or significance of the research. Thus, while these findings provide an overview of the research trajectory, a further qualitative assessment is required to discern the impact and importance of these emerging trends.

Interestingly, the research hotspots in the field of gastrointestinal microbiome and toxicity have gradually shifted from drugs to environmental pollutants. Indeed, since 2018, studies on the correlation between microplastic toxicity and gastrointestinal microbiome have begun to intensify. Microplastics are a common environmental pollutant that can lead to particle toxicity, oxidative stress, and inflammatory responses in organisms (Prata et al., 2020). Jin et al. (2018) found that exposure to two sizes of polystyrene microplastics for 14 days resulted in a significantly decreased abundance of *Bacteroidetes* and *Proteobacteria* in the intestine, while *Firmicutes* significantly increased. Besides, the levels of inflammatory factors IL-1α, IL-1β, and IFN were increased in the gut. Moreover, several studies have confirmed that microplastics can lead to gastrointestinal microbiome dysbiosis and metabolic disorders (Jin et al., 2019; Lu et al., 2019; Qiao et al., 2019). Notably, microplastics may also induce neurobehavioral toxicity through the gut-brain axis, potentially activating neuroactive ligand-receptor interaction and serotonergic synapse-related pathways (Huang et al., 2022). Importantly, Lianguo Chen et al. (2018) found that the toxicity of titanium dioxide nanoparticles and bisphenol A exposure in zebrafish is associated with the gastrointestinal microbiome, with the combined exposure leading to oxidative stress closely related to the ratio of pathogenic Lawsonia and normal metabolic Hyphomicrobium (Chen et al., 2018). Recent research findings indicate that the ingestion of differentially charged nanoplastics leads to the development of inflammatory lesions in the gut, disruption of electron transfer processes, inhibition of energy metabolism during mitochondrial oxidative phosphorylation, oxidative stress, increased expression of pro-inflammatory factors, and disturbances in pathways related to glycolipid metabolism (Xu et al., 2023). Our findings highlight that zebrafish is the predominant model organism in environmental toxicity studies. Their appeal as a crucial animal model in environmental toxicology research stems from several advantages: low maintenance cost, high breeding rate, swift growth, and considerable genetic homology with humans.

The primary target organs in the gastrointestinal microbiome and toxicity research are the gastrointestinal tract, liver, and brain. Studies on the microbiota-target organ axis have flourished in recent years, spurring many researchers to explore toxicology in this field. Zhong et al. (2021) investigated the toxic effects of arsenic trioxide (ATO) on the intestines and liver of ducks. The results demonstrated that ATO could mediate hepatic and jejunal inflammation and pyroptosis through the gut-liver axis and LPS/TLR4/NF-κB signaling pathway (Zhong et al., 2021). Another study found that exposure to perfluorooctanoic acid (PFOA) in mice led to a decrease in intestinal probiotics (including *Lactobacillus* and *Bifidobacterium*), while *Dehalobacterium* and *Pseudomonas* genera were significantly disturbed, which are associated with liver inflammation and oxidative stress. This confirmed that the hepatotoxicity of PFOA might be related to gastrointestinal microbiome dysbiosis (Wang et al., 2021). As previously mentioned, the importance of the gut-brain axis in toxicity research is increasing. Diazinon is an organophosphorus insecticide known to cause neurotoxicity. Studies have found that diazinon disrupts the gut microbiota composition and its metabolic functions in a sex-specific manner (Gao et al., 2017). Sub-chronic and chronic exposure to glyphosate-based herbicides (GBH) induces anxiety and depression-like behaviors in mice and leads to a decrease in the abundance of *Bacteroides*, *Firmicutes*, *Pseudomonas*, and *Lactobacillus* in the mouse gastrointestinal microbiome. Researchers hypothesized that gut dysbiosis might be highly associated with the observed changes in neurobehavior (Aitbali et al., 2018). In a recent study, a correlation between the gut microbiota and two specific organs, the lungs and the brain, has been established. The study found that inhalation of zinc oxide nanoparticles by the lungs can potentially lead to cerebral cortical impairment by disrupting the intricate lung-gut-brain axis (Zhang L. et al., 2023).

Oxidative stress, metabolism, and inflammation are the most reported mechanisms involving gastrointestinal microbiome and toxicity. The gastrointestinal microbiome can induce or exacerbate oxidative stress by metabolizing drugs or environmental pollutants to produce reactive oxygen species or free radicals or influence the host’s antioxidant system. For instance, the oral antiviral brivudine can be metabolized by the host and gastrointestinal microbiome into bromovinyluracil, exhibiting hepatotoxicity. The hepatotoxicity of brivudine may be associated with oxidative stress induced by its metabolic products (Jameson and Hsiao, 2019). Moreover, the gastrointestinal microbiome can induce or modulate inflammatory responses by regulating the host’s immune system or releasing endotoxins or pro-inflammatory cytokines. For example, the gastrointestinal microbiome can indirectly influence an individual’s response to immunotherapy in cancer treatment. Some commensal bacteria in the gut can enhance the antitumor effects of immune checkpoint inhibitors (ICIs), while some pathogenic bacteria may suppress the efficacy of ICIs or increase their toxic side effects (Lu et al., 2022). Nevertheless, certain probiotics can potentially exert a beneficial effect on the toxicity of specific substances. In this respect, the efficacy of *Lactobacillus fermentum HNU312* has been demonstrated in mitigating oxidative damage and behavioral abnormalities induced by chronic lead exposure during early brain development (Zhang Z. et al., 2023). The gastrointestinal microbiome can influence the metabolism of drugs and environmental pollutants in various ways, thereby altering their toxicity (Li et al., 2008; Inamura, 2021).

An important direction for future research in the gastrointestinal microbiome field is exploring the therapeutic potential of manipulating the microbiome to mitigate the harmful effects of toxins. Building upon the existing knowledge of how the gastrointestinal microbiome influences responses to cancer therapies and the effects of environmental pollutants, investigations can focus on interventions such as probiotics, prebiotics, antibiotics, or fecal microbiota transplantation to enhance treatment efficacy and reduce toxicity. In addition, given the complexity of the interactions between the gastrointestinal microbiome and host health, interdisciplinary research approaches will be crucial in advancing our understanding of this field. Collaborations among toxicologists, microbiologists, environmental scientists, and health professionals could significantly advance our understanding of the gastrointestinal microbiome and its role in toxicity.

Despite the insights provided by our bibliometric analysis, this study is not without limitations. This study employed bibliometric analysis to assess research progress on the gastrointestinal microbiome and toxicity. However, it is important to note that bibliometric analysis mainly provides a quantitative assessment of the literature and does not necessarily reflect the quality or impact of the research. It allows for an objective and comprehensive exploration of the literature but does not substitute for a thorough qualitative assessment. Evaluating the quality of bibliometrics should not solely depend on quantitative measures. Instead, it requires an amalgamation of different methods and standards, such as peer review, expert opinion, and societal benefits, among others, to attain a more comprehensive and equitable scientific assessment. We acknowledge that our study was limited to English publications, potentially omitting significant contributions from non-English sources. A more extensive and inclusive approach would entail translating and analyzing scientific literature published in other languages. Furthermore, our study did not include unpublished literature, such as conference presentations, thesis work, and data from ongoing studies, which could also contribute valuable insights. While this literature is an essential part of the scientific discourse, its inclusion often presents practical challenges due to accessibility and verification issues. Nonetheless, future studies should also consider these sources to capture a more comprehensive view of the research landscape. In addition, this study only analyzed publications indexed in the Web of Science Core Collection database, which may not represent the entire body of literature on this topic. Therefore, these results should be interpreted with consideration of this limitation.

## Conclusion

In conclusion, the relationship between the gastrointestinal microbiome and toxicity has become a research hotspot, with a substantial growth in annual publications indicating the global importance of this research field. China and the United States are the core competitive countries in this field. This research has identified the major researchers and institutions involved in this field globally. *Science of the Total Environment* is the most productive and core journal in this research area. Gastrointestinal microbiome and their relationships with metabolism, immune systems, and cancer treatments are considered hot topics, while environmental pollution and health may be the focus of future research. While we have discussed the potential for modifications to the gastrointestinal microbiome as new directions for treating and mitigating toxicity, we acknowledge the complexity of the underlying mechanisms. These complex interactions require further rigorous investigation. In summary, bibliometric analysis can provide valuable insights into the current state of toxicity and gastrointestinal microbiome research, highlight knowledge gaps, and identify potential future developments. These insights can guide future research directions, facilitate collaboration, and ultimately lead to the development of effective gastrointestinal microbiome intervention strategies for preventing and treating toxicity.

## Data availability statement

Publicly available datasets were analyzed in this study. This data can be found here: Web of Science™ (WOS, http://www.webofknowledge.com).

## Author contributions

JD, CL, TJ, and XB analyzed the data, carried out the literature research, and wrote the manuscript. JD, CL, and XZ contributed to interpreting and discussing the results. TJ reviewed and edited before submission. All authors read and approved the final manuscript.
