# Supplementary material for: Global trends and hotspots of gastrointestinal microbiome and toxicity based on bibliometrics
Source: Front Microbiol. 2023 Jul 31;14:1231372. doi: 10.3389/fmicb.2023.1231372 (PMC10425535; doi:10.3389/fmicb.2023.1231372)
Supplement: Supplementary file 1 [file Data_Sheet_1.docx]

**Supplementary Materials**

Figure S1. Core journals identified according to Bradford's Law.

**
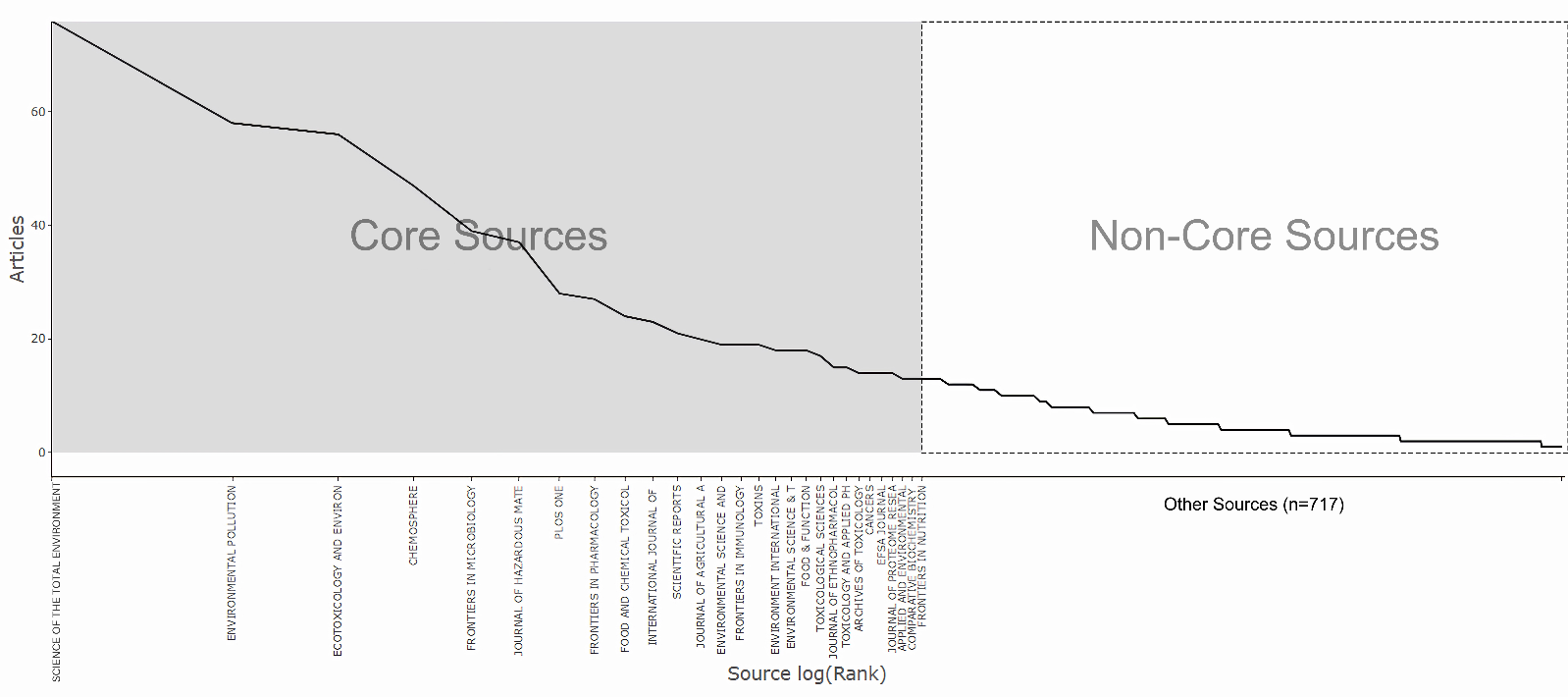
**

Table S1. Search strategy of Web of science Collection.

| #1 | TS=(“Gastrointestinal Microbiome” OR “Gastrointestinal Microbiomes” OR “Microbiome, Gastrointestinal” OR “Gut Microbiome” OR “Gut Microbiomes” OR “Microbiome, Gut” OR “Gut Microflora” OR “Microflora, Gut” OR “Gut Microbiota” OR “Gut Microbiotas” OR “Microbiota, Gut” OR “Gastrointestinal Flora” OR “Gut Flora” OR “Gastrointestinal Microbiota” OR “Gastrointestinal Microbiotas” OR “Microbiota, Gastrointestinal” OR “Gastrointestinal Microbial Community” OR “Gastrointestinal Microbial Communities” OR “Microbial Community, Gastrointestinal” OR “Gastrointestinal Microflora” OR “Microflora, Gastrointestinal” OR “Gastric Microbiome” OR “Gastric Microbiomes” OR “Microbiome, Gastric” OR “Intestinal Microbiome” OR “Intestinal Microbiomes” OR “Microbiome, Intestinal” OR “Intestinal Microbiota” OR “Intestinal Microbiotas” OR “Microbiota, Intestinal” OR “Intestinal Microflora” OR “Microflora, Intestinal” OR “Intestinal Flora” OR “Enteric Bacteria” OR “Bacteria, Enteric”) |
| --- | --- |
| #2 | TS=(toxicity OR toxicities) |
| #3 | #1 AND #2 |

Table S2. Specific parameter information used in Biblioshiny.

| **Option** | **Parameter** |
| --- | --- |
| **Filters** | Default parameters |
| Language | ENGLISH |
| Publication Year | 1980-2022 |
| Document Type | ARTICLE, REVIEW |
| Average Citations per Year | 0-149 |
| Source by Bradford Law Zones | All Sources |
| **Overview** | Default parameters |
| **Sources** | Default parameters |
| Most Relevant Sources | Default parameters |
| Most Local Cited Sources | Default parameters |
| Bradford's Law | Default parameters |
| **Documents** |  |
| Most Global Cited Documents | Default parameters |
| Number of Documents | 10 |
| Measure | Total Citations |
| Cited References |  |
| Most Local Cited References | Default parameters |
| Number of Documents | 10 |
| Field separator character | “;” |
| Words |  |
| Trend Topics |  |
| Field | Keywords Plus |
| Timespan | 1980-2022 |
| Text Editing |  |
| Load a list of terms to remove | No |
| Load a list of synonyms | Yes |
| File Separator | Comma "," |
| File Preview | caco-2 cells <- caco-2 cell |
| Parameters |  |
| Word Minimum Frequency | 5 |
| Number of Words per Year | 3 |

Table S3. Source code for predicting trends and quantities of relevant literature publications using generalized additive models through the MGCV package in R.

| # Load required libraries  library(mgcv)  library(ggplot2)  # Read the data into a data frame  data1 <- data.frame(year = c(1980:2022),count = c(1, 1, 0, 0, 0, 0, 0, 1, 0, 0, 1, 4, 5, 5, 5, 8, 4, 6, 8, 5, 5, 6, 7, 10, 2, 8, 10, 8, 15, 17, 19, 17, 24, 45, 44, 57, 66, 108, 178, 217, 313, 416, 494))  # Fit a GAM model  > model1 <- gam(count ~ s(year), data = data1)  # Plot the fitted curve  > plot(gam(count ~ s(year), data = data1), select = 1)  # Make predictions for 2032  new_data1 <- data.frame(year = 2032)  predict(model1, new_data1)  summary(model1)  Family: gaussian  Link function: identity  Formula:  count ~ s(year)  Parametric coefficients:  Estimate Std. Error t value Pr(>\|t\|)  (Intercept) 49.7674 0.8686 57.3 <2e-16 ***  ---  Signif. codes: 0 ‘***’ 0.001 ‘**’ 0.01 ‘*’ 0.05 ‘.’ 0.1 ‘ ’ 1  Approximate significance of smooth terms:  edf Ref.df F p-value  s(year) 8.918 8.998 1753 <2e-16 ***  ---  Signif. codes: 0 ‘***’ 0.001 ‘**’ 0.01 ‘*’ 0.05 ‘.’ 0.1 ‘ ’ 1  R-sq.(adj) = 0.997 Deviance explained = 99.8%  GCV = 42.166 Scale est. = 32.44 n = 43 |
| --- |

Table S4. Similar keywords were consolidated based on the National Library of Medicine's Medical Subject Headings (MeSH) vocabulary.

| label | replace by | label | replace by |
| --- | --- | --- | --- |
| caco-2 cell | caco-2 cells | diarrhoea | Diarrhea |
| toll-like receptor | toll-like receptors | diarrhea | Diarrhea |
| beta glucuronidase | beta-glucuronidase | induced diarrhea | Diarrhea |
| beta-glucuronidases | beta-glucuronidase | diarrheal disease | Diarrhea |
| antibiotic | Antibiotics | genes | gene |
| amino acid | Amino Acids | 5-flourouracil | 5-fluorouracil |
| amino acids | Amino Acids | aflatoxin b 1 | Aflatoxin B1 |
| amino-acid | Amino Acids | aflatoxin b1 | Aflatoxin B1 |
| amino-acids | Amino Acids | aflatoxin b-1 | Aflatoxin B1 |
| neoplasm | Neoplasms | alzheimer | Alzheimer Disease |
| neoplasms | Neoplasms | alzheimer&apos | Alzheimer Disease |
| tumor | Neoplasms | alzheimer's | Alzheimer Disease |
| tumors | Neoplasms | alzheimer's disease | Alzheimer Disease |
| cancer | Neoplasms | antioxidant | antioxidants |
| colitic cancer | Colonic Neoplasms | anti-oxidant | antioxidants |
| colon cancer | Colonic Neoplasms | bile acid | Bile Acids |
| colon-cancer | Colonic Neoplasms | bile acids | Bile Acids |
| fusarium mycotoxins | Mycotoxins | bile-acid | Bile Acids |
| mycotoxin | Mycotoxins | bile-acids | Bile Acids |
| mycotoxins | Mycotoxins | freshwater | Fresh Water |
| heavy metal | Metals, Heavy | fresh-water | Fresh Water |
| heavy metals | Metals, Heavy | fresh-waters | Fresh Water |
| heavy-metal | Metals, Heavy | protein | Proteins |
| heavy-metals | Metals, Heavy | proteins | Proteins |
| damage | injury | broiler | broilers |
| impairment | injury | broiler chicken | broilers |
| injuries | injury | broiler chickens | broilers |
| biomarker | Biomarkers | broiler chicks | broilers |
| biomarker | Biomarkers | diabetes | Diabetes Mellitus |
| gut barrier | intestinal barrier | diabetes mellitus | Diabetes Mellitus |
| bowel microbiome | Gastrointestinal Microbiome | fecal microbiota transplant | Fecal Microbiota Transplantation |
| enteric bacteria | Gastrointestinal Microbiome | fecal microbiota transplantation | Fecal Microbiota Transplantation |
| enteric bacterial microflora | Gastrointestinal Microbiome | food additive | Food Additives |
| enteric microbiota | Gastrointestinal Microbiome | food additives | Food Additives |
| gut bacteria | Gastrointestinal Microbiome | gastrointestinal tract | Gastrointestinal Tract |
| gut bacterial community | Gastrointestinal Microbiome | bowel | Gastrointestinal Tract |
| gut flora | Gastrointestinal Microbiome | gastrointestinal tract | Gastrointestinal Tract |
| gut fungi | Gastrointestinal Microbiome | gastro-intestinal tract | Gastrointestinal Tract |
| gut micobiome | Gastrointestinal Microbiome | gastrointestinal-tract | Gastrointestinal Tract |
| gut microbes | Gastrointestinal Microbiome | gi tract | Gastrointestinal Tract |
| gut microbial communities | Gastrointestinal Microbiome | gut | Gastrointestinal Tract |
| gut microbial community | Gastrointestinal Microbiome | intestinal tract | Gastrointestinal Tract |
| gut microbiome | Gastrointestinal Microbiome | intestinal-tract | Gastrointestinal Tract |
| gut microbiot | Gastrointestinal Microbiome | intestine | Gastrointestinal Tract |
| gut microbiota | Gastrointestinal Microbiome | intestines | Gastrointestinal Tract |
| gut microflora | Gastrointestinal Microbiome | herbal industries | herbal medicine |
| gut microorganism | Gastrointestinal Microbiome | herbal medications | herbal medicine |
| gut microorganisms | Gastrointestinal Microbiome | herbs | herbal medicine |
| gutmicrobiota | Gastrointestinal Microbiome | herbal medicines | herbal medicine |
| gut-microbiota | Gastrointestinal Microbiome | honey | Bees |
| intestinal bacteria | Gastrointestinal Microbiome | apis mellifera | Bees |
| intestinal biota | Gastrointestinal Microbiome | apis-mellifera | Bees |
| intestinal flora | Gastrointestinal Microbiome | apis-mellifera l | Bees |
| intestinal micro flora | Gastrointestinal Microbiome | apis-mellifera-l | Bees |
| intestinal microbe | Gastrointestinal Microbiome | bee | Bees |
| intestinal microbes | Gastrointestinal Microbiome | bees | Bees |
| intestinal microbial | Gastrointestinal Microbiome | bees apis-mellifera | Bees |
| intestinal microbial community | Gastrointestinal Microbiome | honey bee | Bees |
| intestinal microbiocenosis | Gastrointestinal Microbiome | honey bee phenotypes | Bees |
| intestinal microbiome | Gastrointestinal Microbiome | honey bee queen | Bees |
| intestinal microbiota | Gastrointestinal Microbiome | honey bees | Bees |
| intestinal microflora | Gastrointestinal Microbiome | honeybee | Bees |
| intestinal microorganism | Gastrointestinal Microbiome | honey-bee | Bees |
| intestinal microorganisms | Gastrointestinal Microbiome | honeybees | Bees |
| intestinal mycobiome | Gastrointestinal Microbiome | honey-bees | Bees |
| gut dysbiosis | gut microbiota dysbiosis | microplastics | Microplastics |
| gut microbiome dysbiosis | gut microbiota dysbiosis | microplastic | Microplastics |
| gut microbiota dysbiosis | gut microbiota dysbiosis | nano plastics | Microplastics |
| intestinal dysbiosis | gut microbiota dysbiosis | nanoplastic | Microplastics |
| intestinal microbiota dysbiosis | gut microbiota dysbiosis | nanoplastics | Microplastics |
| intestinalfloraimbalance | gut microbiota dysbiosis | nano-plastics | Microplastics |
| gut microbiome perturbations | gut microbiota dysbiosis | toxicities | toxicity |
| intestinal microbiota disorders | gut microbiota dysbiosis | carassius auratus | Carps |
| herbicide | Herbicides | carp | Carps |
| herbicides | Herbicides | carp ctenopharyngodon-idella | Carps |
| metabolome | metabolomics | carp cyprinus-carpio | Carps |
| metabolomic | metabolomics | common carp | Carps |
| metabonomic | metabolomics | common carps | Carps |
| metabonomics | metabolomics | crucian carp | Carps |
| ms metabolomics | metabolomics | crucian carp(carassiusauratus) | Carps |
| metagenome | Metagenomics | cyprinus carpio | Carps |
| metagenomics | Metagenomics | cyprinus-carpio | Carps |
| metagenomic | Metagenomics | gibel carp | Carps |
| metabolic disorder | metabolic disorders | zebra fish | zebrafish |
| metabolic perturbation | metabolic disorders | zebrafish danio-rerio | zebrafish |
| metabolic-disorder | metabolic disorders | pesticide | Pesticides |
| metabolic-disorders | metabolic disorders | dioxide titanium | titanium dioxide |
| metabolism disorder | metabolic disorders | tio2 | titanium dioxide |
| bacterial-flora | Microbiota | titanium-dioxide | titanium dioxide |
| bacteriome | Microbiota | tio2 nanoparticle | titanium-dioxide nanoparticles |
| microbes | Microbiota | tio2 nanoparticles | titanium-dioxide nanoparticles |
| microbial | Microbiota | titanium dioxide nanoparticle | titanium-dioxide nanoparticles |
| microbiome | Microbiota | titanium dioxide nanoparticles | titanium-dioxide nanoparticles |
| microbiota | Microbiota | titanium-dioxide nanoparticles | titanium-dioxide nanoparticles |
| microbiota&#8211 | Microbiota | radiotherapy | Radiotherapy |
| microbiotne | Microbiota | radiation therapy | Radiotherapy |
| microbome | Microbiota | radiation-therapy | Radiotherapy |
| microflora | Microbiota | tissue | tissues |
| microorganism | Microbiota | lactobacillus plantarum wsj-06 | Lactobacillus plantarum |
| microorganisms | Microbiota | lactiplantibacillus plantarum | Lactobacillus plantarum |
| mycobiome | Microbiota | lactobacillus plantarum | Lactobacillus plantarum |
| coli | Escherichia coli | lactobacillus plantarum jm113 | Lactobacillus plantarum |
| coli (upec) | Escherichia coli | lactobacillus plantarum tw1-1 | Lactobacillus plantarum |
| e coli | Escherichia coli | lactobacillus-plantarum | Lactobacillus plantarum |
| escherichia coli | Escherichia coli | lactobacillus-plantarum ccfm639 | Lactobacillus plantarum |
| escherichia-coli | Escherichia coli | lactobacillus-plantarum ccfm8610 | Lactobacillus plantarum |
| danshen | Salvia miltiorrhiza | lactobacillus-plantarum mon03 | Lactobacillus plantarum |
| salvia miltiorrhiza | Salvia miltiorrhiza | lactobacillus-plantarum ps128 | Lactobacillus plantarum |
| salvia miltiorrhiza bge | Salvia miltiorrhiza | lipid-metabolism | Lipid Metabolism |
| salvia-miltiorrhiza | Salvia miltiorrhiza | lipidmetabolism | Lipid Metabolism |
| prebiotic | prebiotics | lipid metabolism | Lipid Metabolism |
| probiotic | probiotics | fatty liver-disease | Fatty Liver |
| probiotic bacteria | probiotics | fatty liver | Fatty Liver |
